# Efficient generation of recombinant RNA viruses using targeted recombination-mediated mutagenesis of bacterial artificial chromosomes containing full-length cDNA

**DOI:** 10.1186/1471-2164-14-819

**Published:** 2013-11-22

**Authors:** Thomas Bruun Rasmussen, Peter Christian Risager, Ulrik Fahnøe, Martin Barfred Friis, Graham J Belsham, Dirk Höper, Ilona Reimann, Martin Beer

**Affiliations:** 1 DTU National Veterinary Institute, Technical University of Denmark, Lindholm, DK-4771, Kalvehave, Denmark; 2 Institute of Diagnostic Virology, Friedrich-Loeffler-Institut, D-17493, Greifswald-Insel, Riems, Germany

**Keywords:** RNA, Genome, Targeted recombination, Bacterial artificial chromosome, Genetic stability, RNA virus, Pestivirus, Classical swine fever virus

## Abstract

**Background:**

Infectious cDNA clones are a prerequisite for directed genetic manipulation of RNA viruses. Here, a strategy to facilitate manipulation and rescue of classical swine fever viruses (CSFVs) from full-length cDNAs present within bacterial artificial chromosomes (BACs) is described. This strategy allows manipulation of viral cDNA by targeted recombination-mediated mutagenesis within bacteria.

**Results:**

A new CSFV-BAC (pBeloR26) derived from the Riems vaccine strain has been constructed and subsequently modified in the E2 coding sequence, using the targeted recombination strategy to enable rescue of chimeric pestiviruses (vR26_E2gif and vR26_TAV) with potential as new marker vaccine candidates. Sequencing of the BACs revealed a high genetic stability during passages within bacteria. The complete genome sequences of rescued viruses, after extensive passages in mammalian cells showed that modifications in the E2 protein coding sequence were stably maintained. A single amino acid substitution (D3431G) in the RNA dependent RNA polymerase was observed in the rescued viruses vR26_E2gif and vR26, which was reversion to the parental Riems sequence.

**Conclusions:**

These results show that targeted recombination-mediated mutagenesis provides a powerful tool for expediting the construction of novel RNA genomes and should be applicable to the manipulation of other RNA viruses.

## Background

Bacterial artificial chromosomes (BACs) are ideally suited for the stable maintenance of large DNA sequences derived from viral genomes [1]. A considerable number of BAC systems have been established for large DNA viruses; in particular many different herpesvirus genomes have been cloned into BACs (for review see [2]). The first BAC systems using RNA virus cDNAs were described for coronaviruses [3-6] and recently the first BAC containing a full-length cDNA for a negative-stranded RNA virus was described [7]. Similarly, cDNAs corresponding to the full-length genomes of members of the *Flaviviridae* family (Japanese encephalitis virus [8] and Dengue virus [9]) have been inserted into BACs.

BACs containing full-length cDNAs of pestiviruses (also within the *Flaviviridae*), including bovine viral diarrhea virus (BVDV) and classical swine fever virus (CSFV) have recently been established [10,11]. Infectious pestiviruses can be rescued using RNA transcripts derived from these BACs. The pestiviruses have single stranded positive sense RNA genomes, about 12.3 kb in length, which includes a single long open reading frame, encoding a large polyprotein, flanked by 5′ and 3′ untranslated regions (UTRs) that are critical for autonomous replication of the genome [12,13]. The polyprotein is cleaved by cellular and viral proteases into four structural proteins (nucleocapsid protein C, envelope glycoproteins E^rns^, E1 and E2) and eight non-structural proteins (N^pro^, p7, NS2, NS3, NS4A, NS4B, NS5A and NS5B). The availability of genetically defined and stable pestivirus BACs facilitates the functional study of viral proteins or RNA structures and also the development of new marker vaccine candidates. Several CSFV vaccines with marker properties based on chimeric pestiviruses have been developed over the years [14]. In particular, chimeric pestiviruses with substitution of the entire E2 protein have been described [15-17] but also mutants with more subtle modifications, such as the modification of the important TAV-epitope [18] within the CSFV-E2 protein [19,20] are promising marker vaccine candidates.

Manipulation of BACs using traditional cloning procedures can be difficult (e.g. because of a lack of convenient restriction enzyme sites) and thus a range of methodologies that apply bacterial genetics, including homologous recombination (e.g. Red/ET homologous recombineering) within the *E. coli* host, have been developed (for review, see [21]). The use of homologous recombination allows site-directed mutagenesis of BACs [22] and, by employing a counter-selection scheme, specific modifications can be obtained without leaving residual “foreign” sequences [23]. The main advantage of this method is that there are no target limitations (e.g. based on size or location) and no need for suitable restriction sites. The integration of the modified sequence is performed *in vivo* (within *E. coli*) thereby potentially being more accurate than *in vitro* approaches like PCR-based methods. Although *in vitro* cloning approaches based on the use of high-fidelity polymerases for PCR amplification have significantly improved in recent years, the use of *in vivo* approaches should allow a more accurate method of mutagenesis due to the use of the cells own high-fidelity replication system which includes proof reading. Whereas BAC recombination has been commonly used for modifying DNA viruses, there are only very few reports about the use of this technology for RNA viruses [7,24,25].

Here, a generally applicable strategy for the manipulation and rescue of chimeric pestiviruses from BACs is described as a model, and the flexibility of this approach is demonstrated by generating different modifications in the viral cDNA of the new CSFV-BAC, pBeloR26, derived from the modified live vaccine strain “C-strain Riems”. The targeted recombination-mediated mutagenesis described here includes the substitution of the 9 amino acid (aa) linear TAV-epitope (TAVSPTTLR) present in the E2 protein with the corresponding region (TTVSTSTLA) of a heterologous pestivirus (border disease virus, BDV, strain “Gifhorn”) and also the replacement of the entire CSFV E2 protein coding region with the whole E2 coding region from the same BDV, to generate marked vaccine viruses that can be discriminated using specific anti-E2 monoclonal antibodies. The genetic stabilities of both the BAC constructs (within *E. coli*) and the rescued viruses have also been assessed.

## Methods

### Cells and viruses

Porcine kidney (PK15) and sheep fetal thymoid (SFT-R) cells were grown at 37°C (with 5% (v/v) CO_2_) in Dulbecco’s minimal essential medium (DMEM) supplemented with 5% (v/v) pestivirus-free fetal calf serum. Virus from a bait containing the modified live vaccine CSFV “C-strain Riems” (Riemser Arzneimittel AG, Germany) was propagated once in PK15 cells and termed vRiemser. RNA obtained from BDV strain “Gifhorn” [26] was used for amplification of the Gifhorn E2-coding sequence.

### DNA oligonucleotides

Oligonucleotide primers used are listed in Additional file 1: Table S1.

### BAC constructs

The BAC construct, pBeloR26, was constructed using the long RT-PCR method as previously described [11] using RNA derived from the “C-strain Riems”. Briefly, full-length viral cDNAs flanked by *Not*I sites were amplified by long RT-PCR using primers 5′Cstrain_T7_Not1 (which includes a T7 promotor for *in vitro* transcription, a *Not*I site and a region corresponding to the first 44 nt of the genome) and 3′CSFV_Not1 (that contains a *Not*I site and sequence complementary to the 3′-terminal 35 nt of the genome that are conserved among many CSFVs including the C-strain). The product (ca. 12.3 kbp) was digested with *Not*I and inserted into similarly digested pBeloBAC11 (New England Biolabs, GenBank accession U51113). All BACs were modified and maintained in *E. coli* DH10B cells (Invitrogen) grown at 37°C in LB medium containing chloramphenicol (Cam, 15 μg/ml). The electroporation of bacteria was performed in 0.1 cm cuvettes using 1 pulse at 1800 V, 25 μF and 200 Ω in a Gene Pulser Xcell (Bio-Rad). BACs to be used as templates for long PCR or for screening by restriction enzyme digestion were purified from 4 ml overnight cultures of *E. coli* DH10B using the ZR BAC DNA Miniprep Kit (Zymo Research). BACs required for direct genome sequencing were purified from 500 ml cultures using the Large-construct kit (Qiagen).

### Modification of the CSFV cDNA by Red/ET recombination

Modifications to the full-length CSFV cDNA were accomplished in *E. coli* DH10B (streptomycin resistant, Strep^R^) using the Counter Selection BAC Modification Kit (Gene Bridges, Heidelberg, Germany).

The Red/ET recombination involved three steps (*i-iii*). Step *i*) the temperature-sensitive pRedET expression plasmid (Gene Bridges) was introduced into electroporation-competent *E.coli* DH10B cells containing the parental BAC (phenotype Cam^R^, Strep^R^). The pRedET expresses the phage lambda proteins redα, redβ and redγ, under control of the arabinose-inducible pBAD promoter, allowing homologous recombination to occur. Immediately after electroporation, pre-warmed LB medium without antibiotics (1 ml) was added to the cells which were then incubated at 30°C for 1 hour, prior to spreading onto agar plates containing Cam (15 μg/ml) and tetracycline (Tet) (3 μg/ml) and then incubated at 30°C overnight to maintain the pRedET. The presence of the pRedET plasmid (conferring Tet^R^) was verified by visual inspection of BAC-DNA preparations from the Cam^R^/Tet^R^ colonies using agarose gel electrophoresis. Step *ii*) counter-selection marker cassettes with an extra *Not*I site for screening purposes (rpsL-neo, 1325 bp) were amplified by PCR using primers with 30 nt or 50 nt extensions that were homologous to the target site in the BAC using the rpsL-neo plasmid (Gene Bridges) as template and the Phusion hot start II HF DNA polymerase (Thermo Scientific) with cycling conditions as follows: 98°C for 30s, followed by 35 cycles of 98°C for 10s, 60°C for 20s, 72°C for 60s, and 1 cycle at 72°C for 4 min. The PCR products (ca. 1400 bp) were isolated on 1% (w/v) TBE agarose gels and purified using a GeneJET gel extraction kit (Thermo Scientific). Samples (30 μl), from an *E. coli* culture containing pRedET and the parental BAC grown overnight at 30°C in LB media (Cam, Tet), were used to inoculate 1.4 ml of fresh LB media with the same antibiotics to obtain exponentially growing bacteria at 30°C. Red/ET recombination proteins were induced by adding 50 μl of 10% (w/v) L-arabinose (Sigma). The PCR product (200 ng) containing the rpsL-neo cassette was introduced into these bacteria using electroporation (as above). Following electroporation, the cells were grown at 37°C for 70 min (to allow recombination) and then selected on plates containing Cam (15 μg/ml), Tet (3 μg/ml) and kanamycin (Kan, 15 μg/ml) overnight at 30°C to maintain the pRedET. Note, the rpsL cassette confers Streptomycin sensitivity (Strep^S^) onto the resistant DH10B strain and the neo confers Kanamycin resistance (Kan^R^). The correct phenotype (Cam^R^, Kan^R^, Tet^R^, Strep^S^) of the resulting colonies was confirmed by streaking the colonies onto plates containing Cam (15 μg/ml), Tet (3 μg/ml) and Kan (15 μg/ml) and grown at 30°C. Importantly, for the third step, the replacement of the rpsL-neo cassette (using counter-selection), the selected colonies were also streaked onto plates containing Cam (15 μg/ml) plus Strep (50 μg/ml) and shown to be Strep^S^ indicating incorporation of a functional rpsL gene. The structures of the intermediate BACs were verified by restriction enzyme analysis and sequencing around the inserts. Step *iii)* the replacement of the rpsL-neo selection cassettes from the intermediate constructs using linear DNA fragments was achieved through counter-selection and Red/ET recombination. Again, the homologous sequences at the ends of the DNA fragment were used for Red/ET mediated recombination events to replace the rpsL-neo cassette with the sequence of interest. Counter-selection against the rpsL-neo cassette (phenotype Cam^R^, Kan^R^, Tet^R^, Strep^S^) was employed using media containing Cam (15 μg/ml) and Strep (50 μg/ml) to isolate the required derivatives (phenotype Cam^R^ and Strep^R^).

**Figure 1:**
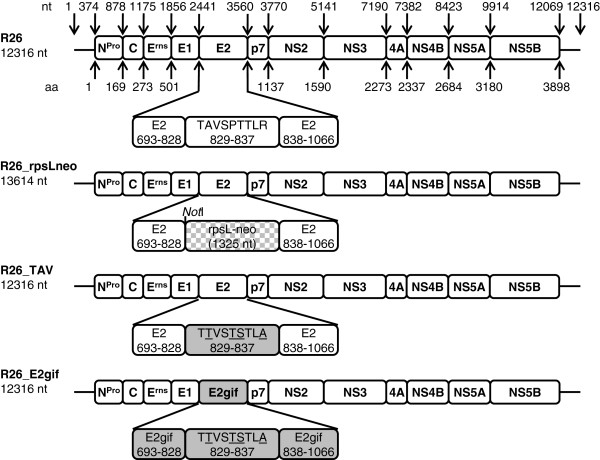
**Schematic representation of the CSFV genome organization and the BACs constructed and used in this study.** Nucleotide (nt) and amino acid (aa) positions within R26 for the 5′ and 3′ termini together with the translational start and stop codons of the polyprotein coding region plus cleavage sites used to make the individual proteins (N^pro^, C, E^rns^, E1, E2, p7, NS2, NS3, NS4A, NS4B, NS5A and NS5B) are indicated. Insertion of the rpsL-neo in place of the TAV-epitope within CSFV E2 for the intermediate construct (R26_rpsLneo) and the subsequent replacement with the TTVSTSTLA sequence (R26_TAV) and the complete substitution of the E2 sequence (R26_E2gif) are shown. Names of BAC constructs begin with “pBelo” and rescued viruses with “v” (e.g. pBeloR26 and vR26). Cell culture passage no. of virus is indicated with “/P” (e.g. vR26/P-4).

Initially, the intermediate construct, pBeloR26_E2rpsLneo (Figure 1), was generated using Red/ET recombination by insertion of the rpsL-neo cassette with an extra *Not*I site for screening purposes which was amplified using primers Criems-TAVfor and Criems-TAVrev (Additional file 1: Table S1) in place of the TAVSPTTLR coding sequence (27 nt). Secondly, the rpsL-neo cassette in this intermediate construct was then replaced using counter-selection Red/ET recombination using a single-stranded oligonucleotide, Riems_TAV_Gifhorn (Additional file 1: Table S1) with the same homology arms as used for the rpsL-neo cassette, to introduce the coding sequence for the BDV “Gifhorn” epitope sequence (TTVSTSTLA). The resulting construct was named pBeloR26_TAV (Figure 1). The initial intermediate construct (with rpsL-neo) was then used to produce the pBeloR26_E2gif construct (Figure 1). For this, the E2 coding sequence was amplified from cDNA prepared from BDV “Gifhorn” RNA using two different primer pairs, one set with 50 nt homology arms (Criems_E2_gifFlong/Criems_E2_gifRlong) and another with 30 nt homologous sequences (Criems_E2_gifF/Criems_E2_gifR).

For generation of BACs with substitution of the entire E2 coding sequences, PCR products consisting of the sequence of interest flanked with homology arms identical to the target area were generated by PCR (as for the rpsL-neo cassette). For making constructs with substitution of shorter sequences (e.g. the TAV-epitope), the recombination was achieved using synthetic single stranded oligonucleotides rather than PCR products. Pre-heating of single stranded oligonucleotides at 95°C for 2 min followed by snap-freezing, prior to electroporation, empirically showed the best results. In each case, the DNA molecules were introduced into *E. coli* containing the BAC derivatives including the rpsL-neo cassettes together with the pRedET plasmid by electroporation as described above. The structures of the modified BACs were verified by restriction enzyme analysis and subsequent full-genome sequencing (see below).

### Rescue of viruses and virus growth curves

BAC DNA (1 μg) was linearized with *Not*I or 1 μl BAC DNA was used as template for long PCR amplification using primers 5′C-strain_T7_Not1 and 3′CSFV (Additional file 1: Table S1). Linearized BACs or PCR products were purified with the GeneJet PCR purification kit (Thermo Scientific) and transcribed *in vitro* using a Megascript T7 kit (Invitrogen). Viruses were rescued from RNA transcripts (1 to 5 μg) by electroporation of porcine (PK15) or ovine (SFT-R) cells essentially as described previously [24]. Cells were analysed using immunofluorescence microscopy (typically after 3 days) for the expression of NS3 and E2 proteins using specific monoclonal antibodies (mAbs), these were anti-NS3 (WB103/105, pan-pestivirus), anti-CSFV E2 (WH211, WH303, both CSFV specific) and anti-BDV E2 (WB166, BVDV/BDV specific) (AHVLA Scientific, United Kingdom) together with Alexa 488 conjugated goat anti-mouse IgG antibody (Molecular Probes, Invitrogen). The nuclei of cells were visualized using DAPI (Vector Laboratories) and images were recorded using a BX63 fluorescence microscope (Olympus). For peroxidase staining, cells were fixed and stained for the presence of pestivirus antigens using biotinylated pig anti-CSFV/BVDV polyclonal IgG followed by avidin-conjugated horseradish peroxidase (eBioscience) as previously described [27]. The same staining procedure was also performed using the anti-E2 mAbs. Samples containing virus-positive cells were passaged onto new cells. Virus growth curves were generated as previously described [24]. Briefly, PK15 or SFT-R cells were infected at a multiplicity of infection (MOI) of 0.1 pfu/cell and grown for three days. At 2, 8, 24, 48 and 72 hours post infection (PK15) or at 3, 12, 24, 48 and 72 hours post infection (SFT-R), cell samples were harvested for virus titration. Cell samples containing virus from each time point were assayed on PK15 or SFT-R cells by limiting dilutions and grown for three days to determine the virus titre (as TCID_50_/ml).

### Genome sequencing

BAC DNAs (5 μg), purified using the Large-construct kit (Qiagen), or PCR products (1 μg) amplified from viral cDNA or from BACs using the long PCR method (as above) were consensus sequenced using a 454 FLX (Roche) or an Ion PGM (Life Technologies). Both Newbler (Roche) and the bwa.bwasw alignment algorithm [28] were used for mapping the reads to the expected sequence. A combination of Samtools [29] and LoFreq SNV-caller [30] was used for downstream single nucleotide variant (SNV) analysis. Finally, clone consensus sequences were aligned using MAFFT in the Geneious software platform (Biomatters).

## Results

### Generation of a BAC containing full-length cDNA corresponding to the modified live vaccine “C-strain Riems”

**Figure 2:**
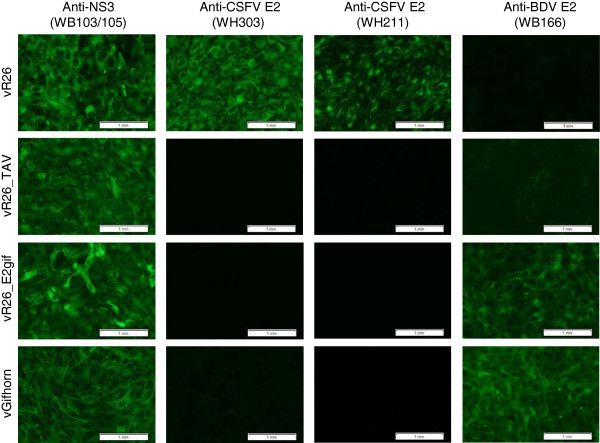
**Antibody reaction patterns of pestivirus infected cells.** SFT-R cells were infected with vR26 and its two derivatives vR26_E2gif and vR26_TAV plus vGifhorn [26]. After 72 h, the cells were fixed and stained with monoclonal antibodies against the NS3 protein (WB103/105, left column), the CSFV E2 protein (WH303 and WH211, middle columns) and the BDV E2 protein (WB166, right column) as indicated and viewed using a fluorescence microscope.

**Figure 3:**
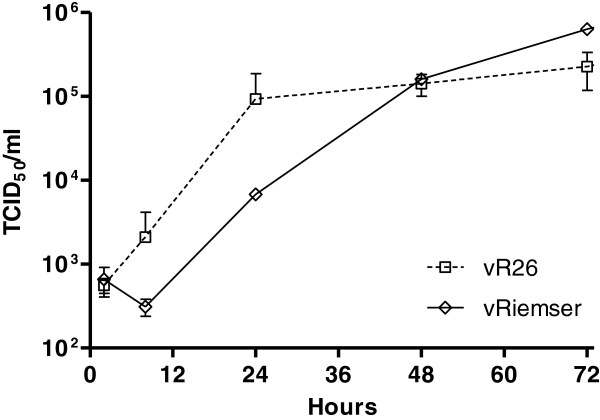
**Growth characteristics of vR26 compared to the parental vRiemser.** The growth of the rescued vR26 and the parental vRiemser strains was evaluated in PK15 cells using an MOI of 0.1 pfu/cell. Virus titers were determined from harvests prepared at 2, 8, 24, 48, and 72 h post infection. Data are represented as mean + SD (n = 3).

BACs containing the full-length cDNA corresponding to the parental vRiemser (“C-strain Riems”) were constructed according to the method described previously for the “Paderborn” strain of CSFV [11]. BACs containing the complete CSFV cDNAs were identified by restriction digest analysis and following linearization by *Not*I, RNA transcripts were produced and electroporated into PK15 cells. This screening resulted in the identification of a BAC containing a cDNA insert of 12316 nt, pBeloR26 (Figure 1), which yielded infectious virus, termed vR26, that could be propagated in SFT-R cells (Figure 2, upper panels) and in PK15 cells (Figure 3). The rescued vR26 displayed higher growth rate at the early stage (about 10-fold difference in virus yield at 24 h) compared to the parental vaccine virus, but after 48 hours similar virus titres were obtained (Figure 3). Full-genome sequencing of the cloned BAC template, pBeloR26, revealed a number of differences throughout the genome when compared to the full-length consensus sequence of the cDNA used for the cloning procedure (see Table 1). These differences are non-representative variants within the cDNA. Overall, the BAC sequence differed from the cDNA sequence in 18 positions, 9 of these lead to predicted amino acid substitutions within the polyprotein; one in each of N^pro^, E^rns^, E1, E2 and NS3 and four amino acid substitutions in NS5B (Table 1). When compared to the published reference sequence (GenBank accession AY259122.1), the pBeloR26 BAC sequence differed at an additional 11 positions, 1 of these lead to a predicted amino acid substitution and there was one large insertion (27 nt) in the hypervariable region of the 3′-UTR (Additional file 2: Table S2).

**Table 1:** Nucleotide and amino acid differences between the consensus cDNA sequences of the parental vaccine virus (vRiemser), the cloned BAC cDNA (pBeloR26) and cDNA of the rescued vR26/P-12

**Region**	**nt position**	**vRiemser (cDNA)**	**pBeloR26 (BAC cDNA)**	**vR26/P-12 (cDNA)**	**aa change (in pBeloR26)**
N^pro^	695	G	A	A	E108K
E^rns^	1427	C	T	T	H352Y
E1	2364	T	C	C	I664T
E2	3068	G	A	A	D899N
NS2	4441	A	G	G	-
NS3	5500	A	G	G	-
	5530	A	G	G	-
	6043	T	C	C	-
	6201	T	C	C	V1943A
NS5A	9589	T	C	C	-
NS5B	10079	A	G	G	M3236V
	10134	A	G	G	K3254R
	10272	A	G	G	K3300R
	10665	G	A	G*	G3431D
3′ UTR	12128	T	A	A	
	12136	T	C	C	
	12137	C	T	T	
	12152	T	A	A	

*Nt position 10665 in vR26/P-12 is reverted from A to G as in the parental cDNA.

### Homologous recombination to obtain CSFV E2 chimeric constructs

To determine the utility of the targeted recombination-mediated mutagenesis system for pestiviruses, two different modifications of the E2 protein coding sequence within pBeloR26 were generated using the Red/ET recombination methodology. Initially, the sequence encoding the linear TAV-epitope (TAVSPTTLR) within the CSFV-E2 was substituted with the sequence encoding the corresponding region (encoding TTVSTSTLA) from the BDV strain “Gifhorn” as described in the Materials and Methods section. More than 90% of the colonies obtained using this procedure contained the required BAC structure as determined by *Not*I digestions. The complete genome sequences of the CSFV cDNA within two selected BACs, designated pBeloR26_TAV have been verified (data not shown). In addition, the complete coding sequence (1119 nt) for the CSFV-E2 protein was substituted by the corresponding sequence from BDV “Gifhorn”. Again more than 90% of the colonies obtained contained the required BAC and the same proportion of correctly recombined BACs was obtained using either 30 nt or 50 nt homology arms. The chimeric BAC was designated, pBeloR26_E2gif and the complete virus genome sequence (cDNA) was verified (data not shown).

### Rescue of modified virus from recombined BACs

After electroporation with RNA transcripts derived from either pBeloR26_TAV or pBeloR26_E2gif a large number of CSFV NS3-positive cells could be observed (data not shown) and chimeric virus stocks, termed vR26_TAV and vR26_E2gif, were generated after further passages in cells. Cells infected with these viruses and with the parental vR26 and vGifhorn strains were all stained with mAbs directed against the NS3 protein (Figure 2). However, in contrast to the parental vR26 virus, the chimeric viruses rescued from the recombined BACs were not recognized by anti-E2 mAbs specific for the CSFV-E2 proteins (Figure 2) and thus, consistent with their structure, displayed the same antibody reaction pattern as vGifhorn. Two different anti-CSFV E2 mAbs, WH211 and WH303, were used for the staining and the latter has been shown previously to target the TAV-epitope [18]. As anticipated, cells infected with either the vGifhorn or with the chimeric vR26_E2gif could be shown to express the “Gifhorn” E2 protein using staining with an anti-BDV mAb (Figure 2). The presence of the BDV epitope TTVSTSTLA in vR26_TAV was insufficient to permit efficient recognition by this anti-BDV mab, although a weak signal was observed in some cells.

### Genetic stability of the BACs in the bacterial host

The BAC constructs pBeloR26 and pBeloR26_E2gif were analysed for the genetic stability of the cDNA to determine the suitability of the BAC vector for maintaining full-length pestivirus cDNAs. *E. coli* DH10B cells containing the BACs were passaged 15 times, by overnight growth, and the complete viral cDNAs within the BACs were sequenced after the 1st and the 15th passage. No mutations were observed within the 12316 nt virus cDNA sequences after this extensive propagation of the BACs in the bacterial host, indicating a highly stable system for the maintenance of complete pestivirus cDNA sequences.

### Genetic stability of viruses rescued from the BACs

**Figure 4:**
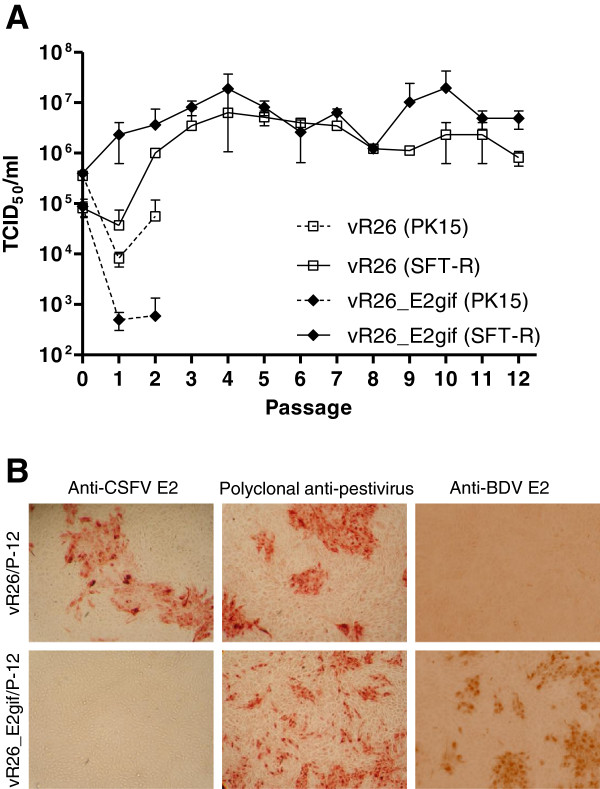
**Characteristics of the vR26 and the chimeric vR26_E2gif in cells. (A)** Virus yield assays were performed during passage (from passage 1 to 12) of vR26 and vR26_E2gif in PK15 and SFT-R cells as indicated. For each passage, cells were harvested after 3 days and the virus titres determined. Data are presented as mean + SD (n = 2). **(B)** Cells (SFT-R) infected with the vR26/P-12 or with the vR26_E2gif /P-12 viruses were stained using a polyclonal anti-pestivirus serum (which recognizes both BDV and CSFV proteins) and with specific mAbs directed against the CSFV E2 (WH211) and BDV E2 (WB166) proteins as indicated.

The viruses, vR26 and vR26_E2gif, rescued from their respective BAC constructs, were also tested for their genetic stability within mammalian cells. Linearized BAC DNA was transcribed *in vitro* and the RNA was electroporated into PK15 cells. Three days after electroporation the cells were stained with the anti-NS3 antibody to detect the presence of replicating virus. Samples containing virus positive cells were passaged onto new cells, this process was repeated for 12 separate passages (each of three days). The virus titre (as TCID_50_/ml) was determined for each passage. Passage of the rescued vR26_E2gif chimeric virus in PK15 cells resulted in rapidly decreasing virus titres and was discontinued after the 2nd passage (Figure 4A). Instead, further passage of this chimeric virus was performed in ovine SFT-R cells (the preferred cell type for BDV) and resulted in much higher titers of the chimeric virus. Virus titers reached more than 10^6^ TCID_50_/ml after the 1st passage and remained stable for 12 passages (Figure 4A). The rescued vR26 was also efficiently propagated on the SFT-R cells but maintained a slightly lower titer than the vR26_E2gif chimeric virus (Figure 4A). To check that the viruses retained their antibody reaction properties (Figure 2) after these passages, cells were infected with viruses from the 12th SFT-R cell culture passage (termed vR26/P-12 and vR26_E2gif/P-12) and stained with a polyclonal anti-pestivirus serum and with specific mAbs directed against the CSFV-E2 and BDV-E2 proteins (Figure 4B). Cells infected with either the vR26/P-12 or the chimeric vR26_E2gif/P-12 were each detected by the polyclonal anti-pestivirus serum as expected. The anti-CSFV-E2 mAb specifically detected cells infected with vR26/P-12 but not cells infected by the chimeric virus containing the BDV-E2 protein (consistent with the results shown in Figure 2). In contrast, the anti-BDV-E2 mAb specifically detected infection by the vR26_E2gif/P-12 and did not recognize cells infected with vR26/P-12. Each result is in accord with the structure of the viruses.

**Figure 5:**
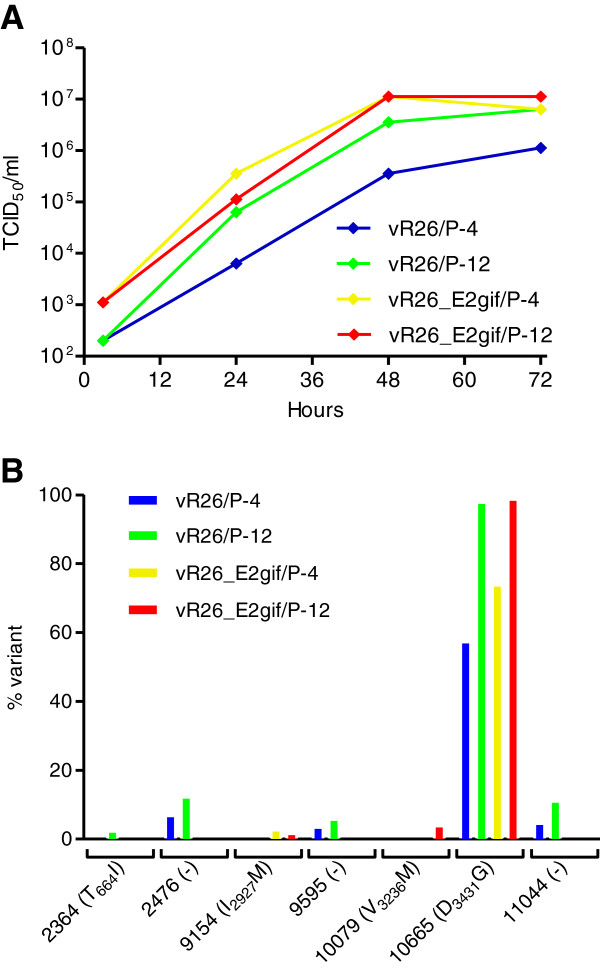
**Growth characteristics and quasispecies distribution of vR26/P-4, vR26/P-12, vR26_E2gif/P-4 and vR26_E2gif/P-12. (A)** Time courses of virus yields after infection of SFT-R cells with vR26/P-4, vR26/P-12, vR26_E2gif/P-4 and vR26_E2gif/P-12. Cells were infected with the viruses (MOI of 0.1 plaque-forming units per cell) and harvested after 3, 24, 48 and 72 h, as indicated, and the virus titres determined (n = 1). **(B)** Single nucleotide variant (SNV) data obtained from cDNA generated from the rescued viruses vR26/P-4, vR26/P-12, vR26_E2Gif/P-4 and vR26_E2Gif/P-12. The bar diagram depicts the quasispecies distribution (as % variant) in the data set from the 454 FLX by Lofreq SNV-caller above 2% in frequency. SNVs (nt position in the cDNA) leading to silent mutations (-) or non-silent mutations (e.g. D3431G) are illustrated.

The 4th passage of vR26 (vR26/P-4) displayed a slower growth rate than the virus obtained after 12 passages (see Figure 5A). It also had a reduced growth rate compared to both the vR26_E2gif/P-4 and vR26_E2gif/P-12. The full-length sequence of pBeloR26 had revealed ten non-silent mutations compared to the reference sequence (AY259122.1) for this virus (Additional file 2: Table S2). Any of these mutations could be responsible for the impaired growth acting alone or in concert. For further investigation of this issue, full length cDNAs prepared from vR26/P-4, vR26/P-12, vR26_E2gif/P-4 and vR26_E2gif/P-12 were deep-sequenced using both the 454 FLX and Ion PGM platforms for comparison and to determine the quasispecies distribution (Additional file 3: Figure S1 and Additional file 4: Figure S2). Sequencing data from both platforms revealed that both the vR26/P-12 and vR26_E2gif/P-12 were close to 100% changed at nt position A10665G compared to the BAC clones (resulting in the predicted amino acid substitution D3431G within the NS5B protein, the RNA-dependent RNA polymerase, see Figure 5B). This adaptation is a reversion back to the consensus cDNA sequence of the parental vaccine virus, vRiemser (Additional file 2: Table S2). Additionally, vR26/P-4 and vR26_E2gif/P-4 already showed evidence for this reversion being present within the population. For vR26/P-4, the level of reversion was 57%, while for vR26_E2gif/P-4 the extent of change was 73% (see Figure 5B).

## Discussion

In this study, we have established the first BAC containing the full-length cDNA of a CSFV vaccine strain. The BAC differed from the parental cDNA sequence in 18 positions leading to 9 aa substitutions (Table 1). The method that has been used for the generation of pBeloR26 is based on full genome amplification of cDNA followed by direct cloning to obtain the BACs [11]. This approach results in cDNA clones that reflect the quasispecies composition of the parental viral RNA and thus it is not guaranteed to obtain cDNA clones corresponding to the consensus sequence of the cDNA used. However, it is possible to correct the mutations using the BAC recombination approach if a consensus clone is needed. To demonstrate the utility of the Red/ET mediated recombination method we have generated a series of modified BACs derived from this CSFV full-length cDNA. These include BACs with substitution of the linear TAV-epitope present in the E2 protein and also BACs with substitution of the complete E2 protein with heterologous pestivirus sequences. We have also used the same approach for a range of different targeted modifications within CSFV BACs including specific deletions and substitutions in the 5′UTR of CSFV [24] and for insertions of heterologous reporter sequences into CSFV replicons [25]. Using Red/ET recombination-mediated mutagenesis for the targeted design, the work can be expedited and focused, in principal, on any sequence within the viral genome and is not dependent on the use of internal restriction sites. The results demonstrate that Red/ET recombination-mediated mutagenesis of pestivirus BAC cDNAs provides a useful tool for advancing the construction of modified pestiviruses.

Cells infected with the parental vR26 virus were recognized by the two anti-E2 mAbs (WH211 and WH303) specific for the CSFV-E2 proteins, in contrast cells infected with the modified viruses vR26_TAV and vR26_E2gif, rescued from the recombined BACs, were not detected by these mAbs. Furthermore, as expected, cells infected with the vR26_E2gif were recognized by the anti-BDV mAb (WB166) whereas no staining was observed with this antibody in vR26 infected cells or in cells with vR26_TAV. The mAb WH303 recognizes the CSFV TAV-epitope [18] and the difference in 4 aa between the TAV-epitope and the corresponding sequence from BDV strain “Gifhorn” is enough to completely abolish the recognition by this mAb. The lack of staining of vR26_TAV infected cells by the WH211 indicated that the TAV-sequence is also important for the epitope recognized by this mAb. Thus, the chimeric pestiviruses, vR26_TAV and vR26_E2gif, containing heterologous E2 sequences can be readily discriminated from the vR26 using specific anti-E2 monoclonal antibodies. These new chimeric pestiviruses represents C-strain based marked vaccine candidates with the characteristics desired for safe and efficacious DIVA vaccines against CSFV. Indeed, vR26_E2gif vaccinated pigs could be efficiently discriminated from C-strain vaccinated pigs and from CSFV infected pigs using CSFV-E2 specific antibody ELISAs (Rasmussen et al., unpublished results).

### Genetic stability in the bacterial host

Nucleotide sequence data for the pBeloR26 showed a number of changes from the published reference sequence for “C-strain Riems”. Some of these differences are present in the cDNA derived from the vaccine stock at a detectable level whereas others may represent low-level variants within the cDNA or errors introduced by the RT-PCR amplification. Full-length sequencing revealed that no changes occurred in the cDNA during extensive propagation in *E. coli* DH10B of the pBeloR26 and the E2-chimeric derivative, pBeloR26_E2gif, indicating a very high stability of these BAC-cloned CSFV cDNAs. This is essential if this system is to be useful for cloning and sequence manipulation, and contrasts with stability problems encountered with conventional plasmids containing full-length pestivirus cDNAs [31]. The stability of these BACs is consistent with previous reports on the stability of BACs containing other viruses of the family *Flaviviridae* in *E. coli*[8,10].

### Genetic stability of rescued viruses

Extensive passaging of the rescued vR26 and the chimeric virus derivative, vR26_E2gif, resulted in a change at nucleotide position A10665G (resulting in the predicted aa change D3431G) within the NS5B coding region. The same reversion to the consensus sequence for the “C-strain Riems” virus was observed in two independent virus populations, derived from independent BACs, and indicates that this substitution is important for efficient growth of the “C-strain Riems” virus in SFT-R cells. In contrast, all other nucleotide positions remained unchanged during the extensive virus propagation including the heterologous E2 sequences (Figure 5B). The glycine (G) residue at position 3431 in the polyprotein is highly conserved amongst CSFV strains; indeed, alignment of CSFV sequences retrieved from GenBank did not reveal any variation at that position (data not shown). Whether this mutation was derived from the cloning procedure or reflects a low-level variant present in the parental vaccine stock is unclear.

## Conclusions

In summary, the present study shows that targeted recombination-mediated mutagenesis of BACs containing full-length CSFV cDNA facilitate manipulation and rescue of chimeric pestiviruses. The system shows high genetic stability of the BAC constructs (within *E. coli*) and the chimeric viruses rescued from the BACs can be efficiently and stably propagated in SFT-R cells and this represents a suitable system for the production of virus stocks for future marked vaccine experiments. The strategies employed in this study have applicability not only for CSFV (and other pestiviruses) but should be adaptable to the study of other RNA viruses.

## Competing interests

The authors declare they have no competing interests.

## Authors’ contributions

TBR, IR and MB conceived the study and developed the approach. TBR, PCR, MBF carried out and optimised the experiments. TBR, UF and DH carried out sequence analyses. All authors contributed to the interpretation of results. Funding was obtained by TBR and GJB. All authors contributed to the drafting and revision of the manuscript. All authors read and approved the final manuscript.

## Supplementary Material

Additional file 1: Table S1 Oligonucleotide primers used in this study. Click here for file

Additional file 2: Table S2 Nucleotide and amino acid differences between the published C-strain “Riems”, the consensus cDNA sequence of the parental vaccine virus (vRiemser) and the cloned BAC cDNA (pBeloR26). Click here for file

Additional file 3: Figure S1 Comparison of vR26/P-4, vR26/P-12, vR26_E2gif/P-4 and vR26_E2gif/P-12 sequence data determined on the Ion PGM and the 454 FLX sequencing platforms. (A)The sequence read distribution per sample is shown as the number of reads for both platforms. (B) The percentage of reads mapped to the pBeloR26 reference sequence by the bwa.bwasw alignment algorithm for all four samples on both platforms is indicated. Click here for file

Additional file 4: Figure S2 Sequence depth per nucleotide position in the genome for vR26/P-4, vR26/P-12, vR26_E2gif/P-4 and vR26_E2gif/P-12 run on the Ion PGM and the 454 FLX sequencing platforms. The horizontally aligned graphs compare the sequencing depth between the sequencing platforms for each sample analyzed by BEDTools [32]. The x-axis depicts the nucleotide position in the viral genome and the y-axis shows the sequencing depth. Click here for file
