# Supplementary material for: Efficient generation of recombinant RNA viruses using targeted recombination-mediated mutagenesis of bacterial artificial chromosomes containing full-length cDNA
Source: BMC Genomics. 2013 Nov 22;14:819. doi: 10.1186/1471-2164-14-819 (PMC3840674; doi:10.1186/1471-2164-14-819)
Supplement: Additional file 1: Table S1 — Oligonucleotide primers used in this study. [file 1471-2164-14-819-S1.docx]

**Additional file 1: Table S1.** Oligonucleotide primers used in this study.

| **Primer:** | **Sequence (5'-3'):** |
| --- | --- |
| 5'Cstrain_T7_Not1 | ATAT*GCGGCCGC*TAATACGACTCACTATAgtatacgaggttagttcattctcgtatAcaCgattggacaaatc |
| 3'CSFV_Not1 | TATA*GCGGCCGC*GGGCCGTTAGGAAATtaccttAgtccaactgtgga |
| 3'CSFV | GGGCCGTTAGGAAATtaccttAgtccaactgtgga |
| Riems_TAV_Gifhorn | GTG CTT TCT ATC TTG TCT GCC CAA TAG GGT GGA CGG GTG TCA TAG AGT GC**A CCA CTG TTA GCA CTA GCA CCC TGG CC**A CAG AAG TGG TAA AGA CCT TCA GGA GAG ACA AGC CCT TTC CGC ACA GAA T |
| Criems_E2_gifF | ATG GCT GTT ACT AGT AAC TGG GGC ACA AGG CCA ATT TGC CTG CAT CGA GAA TTA CA |
| Criems_E2_gifFlong | AGA TCG TGC AAG GTG TGG TAT GGC TGT TAC TAG TAA CTG GGG CAC AAG GCC AAT TTG CCT GCA TCG AGA ATT ACA |
| Criems_E2_gifR | TCA ACA CTA CCT CGC CCT GGC CCA ATG GTA ATG CTG ATG CCA TTT GCT CTG TCA GTA C |
| Criems_E2_gifRlong | GGT GAT TAA GTT CCC TAT CAA CAC TAC CTC GCC CTG GCC CAA TGG TAA TGC TGA TGC CAT TTG CTC TGT CAG TAC |
| Criems-TAVfor | GTG CTT TCT ATC TTG TCT GCC CAA TAG GGT GGA CGG GTG TCA TAG AGT *GCG GCC GC***G GCC TGG TGA TGA TGG CGG GAT CG** |
| Criems-TAVrev | ATT CTG TGC GGA AAG GGC TTG TCT CTC CTG AAG GTC TTT ACC ACT TCT GT**T CAG AAG AAC TCG TCA AGA AGG CG** |

NotI sites are in italics and the T7 promoter is underlined. Nucleotide sequence encoding (TTVSTSTLA) is bold and underlined, whereas the rpsL-neo sequences are displayed in bold.
