# Supplementary material for: Efficient generation of recombinant RNA viruses using targeted recombination-mediated mutagenesis of bacterial artificial chromosomes containing full-length cDNA
Source: BMC Genomics. 2013 Nov 22;14:819. doi: 10.1186/1471-2164-14-819 (PMC3840674; doi:10.1186/1471-2164-14-819)
Supplement: Additional file 2: Table S2 — Nucleotide and amino acid differences between the published C-strain “Riems”, the consensus cDNA sequence of the parental vaccine virus (vRiemser) and the cloned BAC cDNA (pBeloR26). [file 1471-2164-14-819-S2.docx]

**Additional file 2: Table S2.** Nucleotide and amino acid differences between the published C-strain “Riems”, the consensus cDNA sequence of the parental vaccine virus (vRiemser) and the cloned BAC cDNA (pBeloR26).

| Region | nt position | C-strain “Riems”  (AY259122.1) | vRiemser  (cDNA) | pBeloR26  (BAC cDNA) | aa change  (from AY259122.1) |
| --- | --- | --- | --- | --- | --- |
| 5'UTR | 137 | G | A | A |  |
| N^pro^ | 695 | G | G | A | E108K |
| E^rns^ | 1427 | C | C | T | H352Y |
| E1 | 2364 | T | T | C | I664T |
| E2 | 3068 | G | G | A | D899N |
| NS2 | 4111 | C | T (75%) | T | - |
|  | 4441 | A | A | G | - |
| NS3 | 5500 | A | A | G | - |
|  | 5530 | A | A | G | - |
|  | 6043 | T | T | C | - |
|  | 6070 | C | T (83%) | T | - |
|  | 6201 | T | T | C | V1943A |
|  | 6478 | T | C | C | - |
| NS4B | 8305 | A | G (76%) | G | - |
| NS5A | 9328 | C | T (80%) | T | - |
|  | 9475 | T | C (79%) | C | - |
|  | 9589 | T | T (C 8%) | C | - |
| NS5B | 10079 | A | A | G | M3236V |
|  | 10134 | A | A | G | K3254R |
|  | 10272 | A | A | G | K3300R |
|  | 10665 | G | G | A | G3431D |
|  | 11696 | G | A | A | V3775I |
| 3'UTR | 12128 | T | T | A |  |
|  | 12136 | T | T | C |  |
|  | 12137 | C | C | T |  |
|  | 12148 | - | TTTTATTTATTTAGATATTATTATTTA | TTTTATTTATTTAGATATTATTATTTA |  |
|  | 12152 | T | T | A |  |
|  | 12173 | T | C | C |  |
|  | 12185 | C | T | T |  |
