# Supplementary material for: Efficient generation of recombinant RNA viruses using targeted recombination-mediated mutagenesis of bacterial artificial chromosomes containing full-length cDNA
Source: BMC Genomics. 2013 Nov 22;14:819. doi: 10.1186/1471-2164-14-819 (PMC3840674; doi:10.1186/1471-2164-14-819)
Supplement: Additional file 3: Figure S1 — Comparison of vR26/P-4, vR26/P-12, vR26_E2gif/P-4 and vR26_E2gif/P-12 sequence data determined on the Ion PGM and the 454 FLX sequencing platforms. (A)The sequence read distribution per sample is shown as the number of reads for both platforms. (B) The percentage of reads mapped to the pBeloR26 reference sequence by the bwa.bwasw alignment algorithm for all four samples on both platforms is indicated. [file 1471-2164-14-819-S3.docx]

**Additional file 3: Figure S1.** Comparison of vR26/P-4, vR26/P-12, vR26E2gif/P-4 and vR26E2gif/P-12 run on the Ion PGM and the 454 FLX sequencing platforms. (A) The sequence read distribution per sample shown as number of reads for both platforms. (B) The percentage of reads mapped to the pBeloR26 reference sequence by the bwa.bwasw alignment algorithm for all four samples on both platforms.

**A**

**B**
