# Supplementary material for: Efficient generation of recombinant RNA viruses using targeted recombination-mediated mutagenesis of bacterial artificial chromosomes containing full-length cDNA
Source: BMC Genomics. 2013 Nov 22;14:819. doi: 10.1186/1471-2164-14-819 (PMC3840674; doi:10.1186/1471-2164-14-819)
Supplement: Additional file 4: Figure S2 — Sequence depth per nucleotide position in the genome for vR26/P-4, vR26/P-12, vR26_E2gif/P-4 and vR26_E2gif/P-12 run on the Ion PGM and the 454 FLX sequencing platforms. The horizontally aligned graphs compare the sequencing depth between the sequencing platforms for each sample analyzed by BEDTools [32]. The x-axis depicts the nucleotide position in the viral genome and the y-axis shows the sequencing depth. [file 1471-2164-14-819-S4.docx]

**Additional file 4: Figure S2.** Sequence depth per nucleotide position in the genome for vR26/P-4, vR26/P-12, vR26E2gif/P-4 and vR26E2gif/P-12 run on the Ion PGM and the 454 FLX sequencing platforms. The horizontally aligned graphs compare the sequencing depth between the sequencing platforms for each sample analyzed by BEDTools [31]. The x-axis depicts the nucleotide position in the viral genome and the y-axis shows the sequencing depth.
